# The Role of Yoga in Hospitalized COVID-19 Patients: An Exploratory Randomized Controlled Trial

**DOI:** 10.7759/cureus.39320

**Published:** 2023-05-21

**Authors:** Ruchi Dua, Saloni Malik, Ranjeeta Kumari, Manisha Naithani, Prasan K Panda, Amit Saroha, Balram Omar, Monika Pathania, Sudhir Saxena

**Affiliations:** 1 Pulmonary Medicine, All India Institute of Medical Sciences, Rishikesh, Rishikesh, IND; 2 Community and Family Medicine, All India Institute of Medical Sciences, Rishikesh, Rishikesh, IND; 3 Biochemistry, Advanced Center of Continuous Professional Development, All India Institute of Medical Sciences, Rishikesh, Rishikesh, IND; 4 Internal Medicine, All India Institute of Medical Sciences, Rishikesh, Rishikesh, IND; 5 Radiology, All India Institute of Medical Sciences, Rishikesh, Rishikesh, IND; 6 Microbiology, All India Institute of Medical Sciences, Rishikesh, Rishikesh, IND; 7 Medicine, All India Institute of Medical Sciences, Rishikesh, Rishikesh, IND; 8 Radiodiagnosis, All India Institute of Medical Sciences, Rishikesh, Rishikesh, IND

**Keywords:** inflammatory markers, fatigue scores, qol, pranayama, gayatri mantra, covid-19

## Abstract

Introduction

The unpredictable course and sheer magnitude of coronavirus disease 2019 (COVID-19) have sparked a search for novel and repurposed pharmacological interventions. Non-pharmacological interventions may also play a role in the management of this multifaceted disease. This study aimed to evaluate the safety, feasibility, and effect of yoga in hospitalized patients with moderate COVID-19.

Methods

Twenty patients satisfying the inclusion criterion were randomized (1:1 ratio) into Intervention and Control groups. Patients in the intervention arm performed a one-hour yoga session that included pranayama and Gayatri mantra (GM) chant for up to 14 days. Sessions were fully supervised by a trained yoga trainer via an online platform. Patients in both groups received the normal treatment as per national guidelines. Outcome parameters were recorded on the 14th day/end of the hospital stay.

Results

Yoga is safe and feasible in hospitalized patients with COVID-19. The decline of high-sensitivity C-reactive protein (hs-CRP) levels was significantly greater in the Intervention Group. Quality of life (QOL), depression, anxiety, and fatigue severity scale (FSS) showed a decline in both groups with a significant decline observed in FSS scores of the Intervention Group. Median chest X-ray score values, duration of hospital stay, and reverse transcription-polymerase chain reaction (RT-PCR) conversion days were observed to be lower in the Intervention Group but were not significant (p>0.05).

Conclusion

The study found that incorporating pranayama and GM practices in hospitalized patients with moderate COVID-19 pneumonia was safe and feasible. It showed a notable reduction in hs-CRP levels and FSS scores in the Intervention Group, but the study was not powered to detect statistically significant results. Further research with larger sample sizes is needed for conclusive findings.

## Introduction

The coronavirus disease 2019 (COVID-19) pandemic has shaken the world and affected millions globally. With more than 219 million infections worldwide and 4.55 million deaths, it has become one of the most significant infectious disease killers. India alone has witnessed more than 33.9 million Infections and 0.449 million deaths [1]. Apart from various antiviral and anti-inflammatory drugs as part of mainstream treatment, various complementary and alternative therapies have been tried. There are reports in the literature about different herbal medications, Ayurvedic drugs, Indian medicinal plants, and Unani treatments being tried for COVID-19 [2-4]. Meditation, yoga asanas, and pranayama have been proposed for COVID-19 treatment as well as prevention among patients, family members, and healthcare workers [5]. It has been suggested that these complementary approaches may play a role in diseases with inflammatory etiopathogenesis, as these interventions may decrease pro-inflammatory cytokines and upregulate anti-inflammatory markers. Though yoga and pranayama have been evaluated in patients with chronic diseases, their use has been limited in acute or infectious disorders. Even though the Gayatri mantra (GM), one of the most popular and ancient Indian mantras, has conventionally been an integral part of yoga practice, it has been underexplored. COVID-19, apart from being an infectious illness, carries the risk of long-term sequelae and increased burden of psychiatric co-morbidity owing to isolation and uncertainty of the clinical course. This study was designed to evaluate the safety, feasibility, and effect of yoga incorporating pranayama and GM among hospitalized patients with COVID-19 pneumonia in terms of inflammatory markers.

## Materials and methods

Study design

This was a parallel-group prospective randomized controlled trial (RCT) carried out from February 2021 to June 2021 at All India Institute of Medical Sciences (AIIMS), Rishikesh, Uttarakhand, India, and registered on the Central Trial Registry-India (CTRI/2021/02/031036). The study conformed to the principles of the Declaration of Helsinki and was approved by the Institutional Ethics Committee, AIIMS, Rishikesh, India (approval number: AIIMS/IEC/21/43).

Patient selection

All consecutive patients of COVID-19 pneumonia presenting at a tertiary COVID-19 care center, All India Institute of Medical Sciences, Rishikesh, Uttarakhand, India, from February 2021 to June 2021 were screened. Inclusion criteria included (i) age >18 years, (ii) reverse transcription-polymerase chain reaction (RT-PCR)-diagnosed hospitalized cases of COVID-19 patients with moderate pneumonia, (iii) having a smartphone with internet facility, (iv) needing oxygen supplementation, and (v) serum procalcitonin <0.5 ng/ml. Moderate pneumonia was defined as hypoxia with a saturation of 90-94% on room air/dyspnea/respiratory rate >24 breaths/minute as per Indian Council of Medical Research (ICMR) guidelines. The six exclusion criteria were (i) non-consenting adults, (ii) patients needing intensive care, (iii) non-invasive ventilator support or invasive mechanical ventilation or high oxygen requirement (fraction of inspired oxygen >0.40), (iv) patients unable to perform pranayama due to any reason, (v) patients with known psychiatric co-morbidity, or (vi) patients with end-organ damage, e.g., chronic liver disease, chronic renal failure, chronic obstructive pulmonary disease (COPD), persistent or uncontrolled asthma, uncontrolled diabetics, uncontrolled hypertensive, known malignancy, inflammatory bowel disease, arthritis, coronary artery disease, any known chronic inflammatory disease, and COVID-19 patients in severe/critical category as per national guidelines at the time of admission [5].

Sample size power analysis

t tests - Means: Difference between two independent means (two groups)

Analysis: Post hoc: Compute achieved power- given ɑ, sample size, and effect size

Input: Tail(s) = Two

Mean of group 1 = -83.48; Mean of group 2 = 1.82

SD = 126.9; SD = 20.43

Effect size d = 0.93; α err prob = 0.05

Sample size group 1 = 10; Sample size group 2 = 10 

Output: Noncentrality parameter δ = 2.09

Critical t = 2.10

Df = 18

Power (1-β err prob) = 0.5104846

We did not compute the sample size earlier because this is an exploratory RCT; nevertheless, we ran a post hoc power analysis and obtained 51% power for this trial. 

Randomization and blinding

Participants willing to participate in the study and those meeting the inclusion criteria were randomized in a 1:1 ratio to intervention and control groups. The sequence for randomization was computer generated. Group allocation was via the sequentially numbered opaque sealed envelope method. Blinding of subjects was not possible but the outcome assessor and statistician were blinded.

Interventions

Intervention Group

Patients in the experimental group (Intervention Group) performed supervised yoga sessions comprising pranayama and GM, morning and evening for one hour for up to 14 days via online mode. The patient was instructed to sit if possible, or else lie in a comfortable position and take a long deep breath-extended, deep inhalation, and deep exhalation. "Feel positive energy enter your body. When you exhale, then feel all negative energy go outside your body. Give awareness to your inhalation and exhalation. Repeat this practice” [6]. GM chanting was also performed by the patient either sitting or lying as per comfort. If a patient couldn’t chant the mantra, they would listen to the chanting. All patients received the usual treatment as per national guidelines and institutional policy, including (besides others) oxygen inhalation, corticosteroids, antivirals where indicated, and anticoagulants [5].

Control Group

All patients received the usual treatment as per national guidelines and institutional policy, including, among others, oxygen inhalation, corticosteroids, antivirals where indicated, and anticoagulants [5].

Outcomes

The primary outcome was safety, feasibility, and effect on high-sensitivity C-reactive protein (hs-CRP). Secondary outcomes included interleukin 6 (IL6), ferritin, D-dimer, erythrocyte sedimentation rate (ESR), neutrophil-lymphocyte ratio (NLR), chest x-ray (CXR) scores, clinical Improvement in terms of time when declared cured by negative swab on RT-PCR, total duration of hospitalization, final outcome-discharged or died, health-related quality of life (QOL) by St George Respiratory Questionnaire (SGRQ), depression and anxiety scores by Patient Health Questionnaire (PHQ)-9 and Generalized Anxiety Disorder (GAD)-7 scale, and fatigue assessment by Fatigue Severity Scale (FSS) [7-10]. Safety was evaluated by deaths in both groups and complications if any during the intervention (increase in dyspnea or drop in saturation). Feasibility was assessed by the number of patients in the Intervention Group who completed the recommended duration of intervention.

Data collection

Baseline data was collected, including age, sex, level of inflammatory markers, and computed tomography (CT) severity scores. Both groups were followed up to the 14th day/end of the hospital stay, and outcome parameters were recorded. Blood samples for circulating inflammatory markers were collected at baseline and 14th day by venepuncture using plain and ethylenediamine tetraacetic acid (EDTA) tubes, centrifuged for the acquisition of plasma, and stored at −80 °C for subsequent testing. IL-6 levels were determined using chemiluminescence in ADVIA Centaur CP Immunoassay System (Siemens Healthineers, Erlangen, Germany), hs-CRP levels by latex method using a fully automated Beckman Coulter analyzer (Beckman Coulter Inc., Brea, California, United States), ferritin by Latex agglutination in Beckman Coulter fully automated analyzer ((Beckman Coulter Inc.), D-dimer by an immuno-turbidimetric assay using Stago analyzers (Diagnostica Stago S.C.A., Asnières-sur-Seine, France), ESR By MIX-RATE® X20, ELITech Group SAS, Paris, Ile-de-France, France), and NLR by using CELL-DYN Ruby System (Abbott Laboratories, Chicago, Illinois, United States). CXR was performed at baseline and 14th day, and radiological involvement was recorded by an experienced radiologist (with experience of >5 years in radiology) [11-12]. Duration of total hospital stay, outcome, and time of RT-PCR conversion was recorded at the end of hospital stay. Saturation on room air was recorded at baseline and 14th day. All scales (SGRQ, PHQ-9, GAD-7, and FSS) were administered telephonically at baseline and on the 14th day.

Statistical analysis

Data were expressed as mean (standard deviation) as well as median (interquartile range) for quantitative variables. The difference between values at baseline and 14th day/end of hospital stay was calculated for all the quantitative variables. Analysis of data showed non-normal distribution for all the investigated parameters in our study population. A comparison of non-normally distributed numerical data between the groups was performed using Mann Whitney U-test. Categorical variables were reported as proportions and compared using the Chi-square test. A p-value of <0.05 was considered statistically significant. Statistical analysis was performed using IBM SPSS Statistics for Windows, Version 23.0 (Released 2015; IBM Corp., Armonk, New York, United States).

## Results

**Figure 1:**
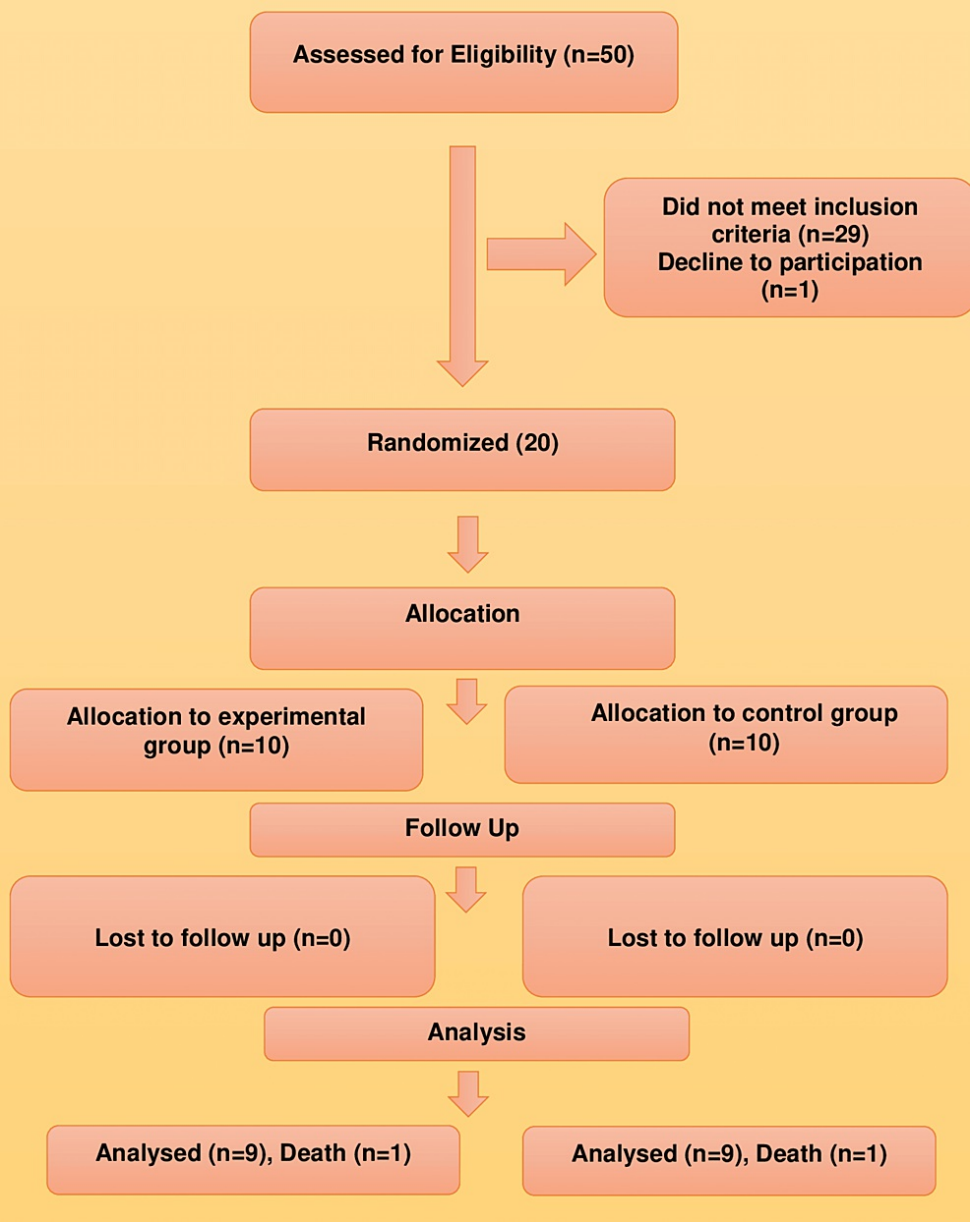
CONSORT diagram CONSORT: Consolidated Standards Of Reporting Trials

Between February 2021 and April 2021, 50 consecutive patients with COVID-19 were screened, out of which 20 patients fulfilling the inclusion criterion were enrolled and randomly allocated to the intervention group or the control group (Figure 1). Baseline demographic data, level of inflammatory markers, other hematological investigations, CT severity index, CXR, SGRQ, PHQ-9, GAD-7, and FSS scores were comparable between the two groups except for hs-CRP levels (Table 1). Hs-CRP levels at baseline showed a significant difference between these groups, with median levels higher in the intervention group (Median 111.72, IQR 38.66-220.45) versus the control group (24.86, 13.24-56.15). Table 2 shows that the two groups were comparable in terms of the proportion of categorical variables such as gender, educational qualification, and co-morbidities like diabetes mellitus and hypertension at baseline.

**Table 1:** Comparison of baseline characteristics of continuous variables between two groups of patients HbA1C: glycosylated hemoglobin; hs-CRP: high-sensitivity C-reactive protein; IL6: interleukin-6; LDH; lactate dehydrogenase; ESR: erythrocyte sedimentation rate; CBC: complete blood count; TLC: total leukocyte count; NLR: neutrophil-to-lymphocyte ratio; RBC: red blood cell;Hb: hemoglobin; HCT: hematocrit; CXR: chest X-ray; CTSS: computed tomography severity scale; SGRQ: St. George’s Respiratory Questionnaire; PHQ-9: Patient Health Questionnaire -9; GAD-7: Generalized Anxiety Disorder scale 7; FSS: Fatigue Severity Scale

Baseline characteristics (Units)	Intervention Group (n=10)					Control Group (n=10)					p-value
	Mean	Std. Deviation	Median	Percentiles		Mean	Std. Deviation	Median	Percentiles		
				25	75				25	75	
Age (years)	55.9	9.83	55	47.5	66.25	53.8	11.87	55	39.75	64.75	0.67
HbA1C (mmol/mol)	6.05	5.78	6.7	0	9.38	7.81	4.71	7.9	5.8	12	0.63
hs-CRP (mg/L)	148.26	138.71	111.72	38.66	220.45	56.69	89.98	24.86	13.24	56.15	0.02
IL6 (pg/ml)	25.78	29.78	15.3	4.92	39.45	52.96	43.4	50.1	11.91	75.63	0.15
Ferritin (µg/L)	571.32	435.87	470.13	162.53	1086.27	790.52	682.78	507.75	284.96	1516.73	0.44
D-dimer (mcg/mL)	2.92	6.03	0.83	0.63	1.78	3.32	5.9	1.43	0.94	2.56	0.32
ESR (mm/hr)	28.7	7.69	30	23	35	28.9	10.05	30	19	35.75	0.90
CBC TLC (cells/ mm^3^)	8.42	3.33	8.33	5.9	10.46	8.49	4.19	6.62	5.36	12.47	0.85
Neutrophil (%)	79.91	9.71	82	72.91	88.18	81.08	6.07	81.26	76.2	86.44	0.97
Lymphocyte (%)	13.18	7.28	9.6	7.83	18.82	12.21	6.25	11.38	6.66	19.95	0.91
NLR	8.07	4.47	8.93	3.88	10.54	9.08	6.2	7.21	3.83	12.05	0.91
RBC count (million/mm3)	4.62	1.06	4.35	3.91	5.03	4.47	0.49	4.32	4.02	4.92	0.74
Hb (gm/dL)	11.99	1.54	11.69	10.6	13.54	11.85	1.27	12.02	10.85	12.87	0.85
HCT (%)	39.53	4.43	40.92	35.26	42.85	39.91	4.63	40.04	36.35	44.44	0.97
Platelets (thousand/mm^3^)	162.91	53.03	132.7	127.78	218.63	181.45	68.84	159.45	117.75	247.65	0.80
CXR score	7.9	4.09	7.5	3.75	12.25	8.3	4.74	8.5	3	11.25	0.96
CTSS	21.9	8.92	21	17.25	28.25	27.4	5.4	26.5	25.25	31.25	0.08
SGRQ	56.17	7.95	53.94	50.25	61.28	58.67	10.44	55	52.72	62.81	0.54
PHQ-9	15.2	3.58	17.5	10.75	18	15	3.97	14	12	17.25	0.69
GAD-7	11.6	2.22	12	9.75	12.5	11.4	2.37	11.5	9.75	12.5	0.84
FSS	23.5	4.09	24	21.75	27	22.6	5.72	21.5	17.75	27.5	0.57

**Table 2:** Comparison of baseline characteristics of categorical variables between two groups

Baseline characteristics		Intervention Group		Control Group		Test statistic	p-value
		n=10	%	n=10	%		
Gender	Male	6	60.0	5	50.0	0.202	1.00
	Female	4	40.0	5	50.0		
Educational qualification	1-10	3	30.0	4	40.0	0.219	0.64
	≥12	7	70.0	6	60.0		
Hypertension	Yes	0	0	3	30.0	3.52	0.21
	No	10	100	7	70.0		
Diabetes mellitus	Yes	4	40.0	6	60.0	0.80	0.37
	No	6	60.0	4	40.0		

Safety and feasibility

No patient in the Intervention Group had a significant increase in dyspnea or drop in saturation during the intervention showing the safety of the intervention. One death was reported in each group. All the surviving patients (9/9) in the Intervention Group were able to complete the prescribed intervention, which was directly supervised by a yoga expert, showing its feasibility in indoor settings among moderate cases.

Inflammatory markers

We compared the two groups for changes in different parameters between enrolment and the 14th day by subtracting the values at baseline from those on the 14th day. It was observed that the absolute mean difference of hs-CRP values was significantly higher in the intervention group with values on the 14th day being lower than that at baseline (Mean -83.48 (SD 3.56)); the values of hs-CRP increased on the 14th day in the Control Group (Mean 1.82 (SD 20.43)). The median values of difference of baseline and 14th-day values of hs-CRP in both groups showed a decline. Still, the decline was significantly higher in the Intervention Group (Median -27.8 (IQR -134.65 to -5.9)) as compared to the Control Group (-0.49, (-9.19 to -21.77) (p-value 0.008) (Table 3). Differences between baseline and 14th-day values for the rest of the inflammatory markers did not differ significantly between the two groups. Saturation at baseline and 14th day did not differ significantly between the two groups.

**Table 3:** Comparison of change in various characteristics at discharge among two groups of patients hs-CRP: high-sensitivity C-reactive protein; IL6: interleukin-6; LDH: lactate dehydrogenase; ESR: erythrocyte sedimentation rate; SGRQ: St. George’s Respiratory Questionnaire; PHQ-9: Patient Health Questionnaire -9; GAD-7: Generalized Anxiety Disorder scale 7; FSS: Fatigue Severity Scale; SpO2: oxygen saturation

Change in	Intervention Group (n=10)					Control Group (n=10)					Mann-Whitney U test statistic	Exact Sig. [2*(1-tailed Sig.)]
	Mean difference	Std. Deviation	Median difference	Percentiles		Mean difference	Std. Deviation	Median difference	Percentiles			
				25	75				25	75		
hs-CRP (mg/L)	-83.48	126.9	-27.8	-134.65	-5.9	1.82	20.43	-0.49	-9.19	21.77	13.0	0.008
IL6 (pg/ml)	-4.85	5.41	-1.7	-9.1	-1	-20.77	22.96	-6	-42.5	-2.08	28.0	0.287
Ferritin (µg/L)	97.27	635.07	-9.66	-212.83	17.68	-387.3	748.25	-167.71	-1079.1	-43.07	30.0	0.143
D-dimer (mcg/mL)	2.72	8.66	-0.01	-0.36	5.47	1.52	6.5	-0.06	-0.98	1.37	45.0	0.739
ESR (mm/hr)	-3.8	6.65	-2	-3.25	-2	-0.9	10.89	-2	-4	-0.25	49.5	0.97
SGRQ	-9.31	9.05	-8.87	-13.07	-2.72	-5.34	2.55	-4.84	-7.5	-3.12	32.0	0.489
PHQ-9	-6.22	2.95	-7	-8.5	-3	-3.67	2.78	-3	-6.5	-1.5	20.5	0.081
GAD-7	-5.44	3.17	-4	-9	-2.5	-3.33	1.66	-4	-5	-1.5	26.5	0.23
FSS	-8.33	4.42	-8	-12	-4.5	-3.44	3.4	-2	-6.5	-2	16.5	0.033
SpO2 (%)	8.78	3.56	8	6	10.5	9.22	3.67	10	6.5	12.5	34.0	0.589

Hematological parameters

The median difference in baseline and 14th-day values of NLR in the Intervention Group was higher (median 3.5 (IQR -0.25 to 11.5)) as compared to the control group (median 1.5 (IQR -4.75 to 9.5)), but it did not reach the level of significance.

Radiological parameters

Median scores of CXR on the 14th day in the Intervention Group showed lesser severity (median 6 (IQR 3 and 10)) as compared to the Control Group (median 7.5 (IQR (3.0 and 10.0)); however, it did not reach the level of significance.

SGRQ scores

The absolute value of median differences in baseline and 14th-day SGRQ scores in the Intervention Group (median -8.87 (IQR -13.07 and -2.72)) was higher than that in the Control Group (median -4.84 (IQR -7.5 and -3.12)) indicating a greater fall in the Intervention Group; however, no significant differences in baseline and 14th-day SGRQ scores were seen. 

PHQ-9 scores

A greater decline in PHQ score was reported in the Intervention Group (median -7 (IQR -8.5 and-3)) as compared to the Control Group (median -3 (IQR -6.5 and -1.5)), but the two groups did not differ significantly.

GAD-7 scores

GAD-7 scores with median -4 (IQR -2.5-9) in the Intervention Group and median -4 (IQR -5 and -1.5) in the Control Group are seen and the difference is not significant between the two groups.

FSS scores

Change in fatigue scores was significantly higher in the Intervention Group (median -8 (IQR -12 and -4.5)) as compared to the Control Group (median -2 (IQR -6.5 and -2) (p-value = 0.033). 

Outcomes

Though the duration of hospital stays and time for RT-PCR conversion were lower in the Intervention Group as compared to corresponding values in the Control Group, the differences between the two groups did not reach the level of statistical significance. One death was reported in each group with no significant difference in outcome between the two groups (Table 4).

**Table 4:** Comparison of various characteristics at discharge among two groups of patients CXR: chest X-ray; NLR: neutrophil-to-lymphocyte ratio; RT-PCR: reverse transcription-polymerase chain reaction

Characteristics at discharge (Units)	Intervention Group (n=10)					Control Group (n=10)					Mann-Whitney U test statistic	p-value
	Mean	Std. Deviation	Median	Percentiles		Mean	Std. Deviation	Median	Percentiles			
				25	75				25	75		
CXR score	6.5	3.06	6.0	3.0	10.0	6.70	3.19	7.5	3.0	10.0	48.00	0.896
Duration of hospital stay	10.1	2.88	10	7.75	12.25	12.2	6.01	11	7.75	17.25	41.5	0.538
NLR	13.82	12.11	8.05	4.58	24.33	10.9	9.44	7.86	3.47	16.04	44.5	0.7
RT-PCR conversion days	9.3	4.9	9	6	13	9.8	7.67	7.5	5	16.25	48.0	0.892

## Discussion

This study was proposed as an exploratory pilot study to see the feasibility, safety, and effect of yoga (pranayama and GM) among patients with COVID-19 pneumonia. Pranayama is practically possible in bed-confined COVID-19 patients while GM, being one of the most popular and ancient mantras, is an integral part of yogic practice and so was clubbed together as part of this study.

There is a lack of well-designed trials regarding the duration, frequency, and effect of GM. Limited studies available indicate an effect on QOL, attention span, and anxiety levels possibly by action on various neurological centers as demonstrated by electroencephalography and functional magnetic resonance imaging on meditation naïve subjects. Its effects on inflammatory markers have been an underexplored area and the exact pathways of action remain unknown [11-12]. A quasi-experimental study on 46 post-stroke subjects compared additional treatment with GM and the emotional freedom technique for seven consecutive days, administered once a day with a standard hospital rehabilitation program, and showed higher QOL scores [13]. Another study with crossover design among 60 healthy school students, showed that GM improved attention spans [14]. An Indonesian study based on pre-post-test and quasi-experimental design showed significant effects with GM administered for five weeks in decreasing anxiety among 34 elderly people [15].

More data is available regarding pranayama and its effects. Pranayama is the traditional technique of slow and rhythmical breathing. It can increase parasympathetic tone, decrease sympathetic activity, and improve cardiovascular and respiratory functions. It significantly decreased states of anxiety and negative affect and its effects can be via modulation of the activity of brain regions involved in emotional processing, particularly the amygdala, anterior cingulate, anterior insula, and prefrontal cortex [16].

In a systematic review on the beneficial health effects of pranayama in respiratory, cardiovascular, and malignant diseases, the duration ranged from days to six months, with variation in duration per day from five minutes a day to 45 minutes twice a day [17]. Reduction in the frequency of attacks, severity, medication requirement, improvement of physiological variables, and improved QOL was reported in bronchial asthma. In patients with COPD, symptom, activity, and impact scores were improved. QOL improvement has been noted in cancer patients, with enhanced emotions, sleep, reduced fatigue, and anxiety. It can also impact the immune levels and antioxidant levels in cancer patients. In patients with hypertension pulse rate, systolic and diastolic pressures were reduced with Pranayama. So available evidence indicate both physiological and psychological benefits of pranayama in chronic disorders, especially cardiorespiratory and malignant diseases. Evidence regarding its role in acute disorders is limited. In a double-blind, RCT of 80 eligible patients undergoing angiography, the experimental group performed five-minute Sukha pranayama exercises before the procedure. The authors found significant changes in anxiety scores in the intervention arm [18,19].

Very few studies have tried to see its effect on COVID-19. A study among healthy individuals during COVID-19 lockdown using yogic breathing intervention found it to increase pulmonary reserve and decrease anxiety [19]. In another study among healthcare workers, the authors explored the feasibility of virtual yoga-based breathwork and meditation during the COVID-19 pandemic and concluded that yoga-based breathing practices were feasible and acceptable [20,21]. Others have proposed that pranayama via nitric oxide pathway and increased carbon dioxide by extended exhalation and alkaline pH prevents coagulopathies and morbidity due to COVID-19 [22].

Isolation and alienation from family members apart from the disease itself lead to increased psychiatric manifestations in COVID-19. Pranayama in various forms has been evaluated and found to influence psychiatric manifestations like anxiety [22-24]. A systematic review of these complementary and alternative medicine (CAM) interventions in COVID-19, which included acupuncture, traditional chinese medicine (TCM), relaxation,and qigong concluded that CAM significantly improved various psychological symptoms, including depression, anxiety, stress, sleep quality, negative emotions, quality of life, and alleviate physical symptoms, including chest pain, and respiratory function [24]. Similarly, we found changes in various domains, including SGRQ, FSS, PHQ-9, GAD -7, and differences in duration of hospital stay, and RT-PCR conversion though only changes in fatigue scores were significant (p<.05).

The exact mechanism through which complementary therapies act is still not known. A review including 26 trials that examined the effects of Tai chi, qigong, meditation, and yoga interventions on inflammatory outcomes reported mixed evidence that the mind-body therapies (MBTs) led to alterations in CRP levels [25]. Half of the studies showed decreases (or attenuated increases) of CRP in the intervention group more marked in patients with higher baseline levels, and the other half of the trials showed no significant change. The evidence for the effects of MBTs on IL-6 and other inflammatory markers was also mixed, with the majority showing no change. However, the trials that yielded non-significant results for inflammatory markers did show beneficial effects on symptoms and other outcomes.

We found a more significant decline in hs-CRP levels in the Intervention Group despite higher baseline values consistent with few studies on yoga in the systematic review [24]. Changes in remaining biomarkers were not significant between the two groups. Wide variability in values can be explained byvariations in levels of various inflammatory biomarkers in different phases of COVID-19 disease and its inherent heterogeneity. Other hematological parameters like NLR showed better recovery in the intervention group along with lesser time for RT-PCR conversion and hospitalization but were not significant. Saturation levels at baseline and discharge were not statistically different between the two groups. Radiological involvement on the 14th day did not differ significantly between the two groups. Improvement in SGRQ scores, the decline in levels of PHQ-9, GAD-7, and FSS on the 14th day was more in the Intervention Group, but it did not reach the level of statistical significance except for a change in FSS (p<.05) in the Intervention Group. An association between fatigue and inflammatory marker has been proposed in studies on cancer survivors [26]. Hs-CRP may serve as a potential mechanism explaining the impact of our intervention on fatigue scores as age and gender were not significantly different between the two groups.

COVID-19 is associated with a substantial mental burden both due to the uncertain long-term course of the disease as well as alienation from family members during the period of isolation [27]. GM and pranayama may serve as a low-cost strategy to mitigate its effects to some extent in addition to the limited pharmacological options available. We have tried to evaluate pathways through which GM and pranayama work. Our study provides the effect of these interventions to be mediated via inflammatory markers like hs-CRP though larger studies would be needed to exactly elucidate precise mechanisms of action.

To the best of our knowledge, there are no other studies yet published on the effect of GM and pranayama in hospitalized COVID-19 patients. It is under-evaluated in acute disorders like COVID-19 with known long-term sequelae. Using interventions like yoga (GM and pranayama) may aid in alleviating multifaceted presentations of COVID-19, especially fatigue scores via inflammatory pathways like hs-CRP. It is both feasible and can be safely performed in hospitalized patients with moderate respiratory involvement.

Limitations

The small sample size is the most significant limitation of this study as it is an exploratory RCT. Blinding of patients was not possible due to study design and space constraints, though the outcome assessor and the statistician were blinded.

## Conclusions

Based on the findings of this study, it was observed that incorporating pranayama and GM practices in hospitalized patients with moderate COVID-19 pneumonia demonstrated safety and feasibility. Additionally, the intervention resulted in a more substantial reduction in hs-CRP levels and FSS scores. However, it is important to note that the study was not powered to detect statistically significant results. Therefore, the observed outcomes should be interpreted with caution, and further research with larger sample sizes is necessary to establish the significance of these findings.
